# Insights into the glioblastoma tumor microenvironment: current and emerging therapeutic approaches

**DOI:** 10.3389/fphar.2024.1355242

**Published:** 2024-03-08

**Authors:** Dev Kumar Tripathy, Lakshmi Priya Panda, Suryanarayan Biswal, Kalpana Barhwal

**Affiliations:** ^1^ Department of Physiology, All India Institute of Medical Sciences (AIIMS), Bhubaneswar, India; ^2^ Department of Human Genetics and Molecular Medicine, Central University of Punjab, Bathinda, India

**Keywords:** glioblastoma, tumor microenvironment, angiogenesis, immunotherapy, blood-brain barrier, therapeutic approaches

## Abstract

Glioblastoma (GB) is an intrusive and recurrent primary brain tumor with low survivability. The heterogeneity of the tumor microenvironment plays a crucial role in the stemness and proliferation of GB. The tumor microenvironment induces tumor heterogeneity of cancer cells by facilitating clonal evolution and promoting multidrug resistance, leading to cancer cell progression and metastasis. It also plays an important role in angiogenesis to nourish the hypoxic tumor environment. There is a strong interaction of neoplastic cells with their surrounding microenvironment that comprise several immune and non-immune cellular components. The tumor microenvironment is a complex network of immune components like microglia, macrophages, T cells, B cells, natural killer (NK) cells, dendritic cells and myeloid-derived suppressor cells, and non-immune components such as extracellular matrix, endothelial cells, astrocytes and neurons. The prognosis of GB is thus challenging, making it a difficult target for therapeutic interventions. The current therapeutic approaches target these regulators of tumor micro-environment through both generalized and personalized approaches. The review provides a summary of important milestones in GB research, factors regulating tumor microenvironment and promoting angiogenesis and potential therapeutic agents widely used for the treatment of GB patients.

## 1 Introduction

The most common CNS tumors in adults are meningioma (15%), glioblastoma (GB) (20%), and metastatic tumors (40%) (Bikfalvi et al., 2023). According to the World Health Organization (WHO) classification, astrocytomas are characterized according to their histopathology and molecular patterns (Table 1). Glial fibrillary acidic protein (GFAP) loss indicates malignancy and characteristic undifferentiated tumor cells. Molecular characterization includes IDH-mutant and IDH-wild type. GB is referred to as an IDH-wildtype astrocytoma with microvascular proliferation, high stemness, invasiveness and predisposition to necrosis (Louis et al., 2021). GB is mostly located in the frontal and temporal lobes of the brain and rarely found in the brainstem, cerebellum and spinal cord. It remains an irrepressible disease with a median survival of 15 months (Chang et al., 2016). Based on the CBTRUS Statistical Report: Primary Brain and Other Central Nervous System Tumors Diagnosed in the United States in 2012–2016 report incidence rate of GB is 3.22 per 100,000 persons in the United States with a median age of 64 years (Ostrom et al., 2019). In an Indian scenario, gliomas comprise 38.7% of tumors of the central nervous system (CNS), of which high-grade (grades III and IV) types constitute 59.5% and low-grade (grades I and II) account roughly 33.1%. In India, there were 23,000 cases of GBM incidence and 49000 widespread cases in 2016 (Rana et al., 2019).

**TABLE 1:** Classification of astrocytomas according to WHO Guidelines (Louis et al., 2021).

Astrocytoma	WHO Grade 2	WHO Grade 3	WHO Grade 4	Glioblastoma
WHO Grade				
**His topathology**	Mild nuclear atypia, dense fibrillar background. Elongated nuclei, hyperchromatic irregular. Microcyst formation in the tumor storma	Cellularity, higher nuclear atypia, increased mitotic activity, round nuclei	Microvascular proliferation, accumulation of hyperplasticity endothelial cells, necrosis	Microvascular proliferation or necrosis. Lesions show higher degree of cellular and nuclear polymorphism, multinucleated giant cells. Astrocytic nature of the neoplasms
**Criteria**	IDH mutant, deletions of CDKN2A and CDKN2B absent	IDH mutant, loss of ATRX mutation , tp53 mutation	IDH mutant, homozygous deletion of CDKN2A and/or CDKN2B	IDH-wildtype, TERT promoter mutation, EGFR gene amplification, +7/-10 chromosome copy- number alterations
**Proliferation index**	Kr-67 proliferation index is 4%.	Ki-67 proliferation index ranges from 4- 10%	Vary considerably, not associated with survival	Elevated Ki-67 proliferation index
**Molecular alterations**	1p/19q intact	1p/19q intact	1p^1^19q intact	1p/19q intact?
**Methylation status**	G-CIMP high	G-GIMP high	0-CIMP high low?	G-CIMP low

GB is very uncommon in the pediatric population (Singla et al., 2021) and is found to be more prevalent in adult males (66.6% more) as compared to females. The gender-based age distribution in glioblastoma patients shows 70.5% males in the age group below 18 years, 69.7% males in the age group between 19 and 50 years and 72.8% males above 50 years (Singh et al., 2021). The number of cases is expected to rise with the rise in the elderly population, particularly in developing countries like the United States. Notwithstanding rigorous therapeutic interventions that include surgical resection, radiation, and concurrent chemotherapy, the prognosis of GB continues to be poor due to its recurrence and poor survival outcomes.

Despite advances in the understanding of cancer biology, the heterogeneous makeup of the tumor microenvironment continues to be a challenge for treatment and management of GB across all age groups. The current experimental models GB cancer models fail to effectively mimic the tumor microenvironment comprising of tumor organismal milieu and niche that plays a key role in promoting and sustaining GB.

### 1.1 Milestones in glioblastoma research

**FIGURE 1:**
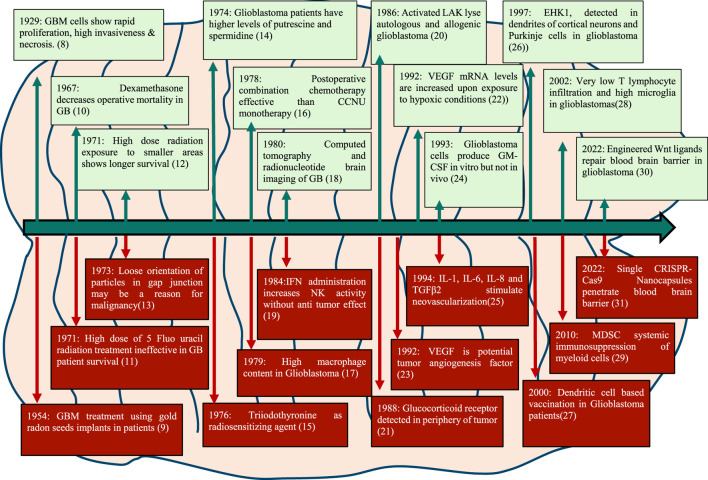
Major milestones in the management and treatment of GB since 1929.

Seminal publications in 1929 described the tissue structure of glioblastoma revealing its characteristic features like necrosis, abundant mitotic figures and invasive morphology with the large nucleus. Some important milestones in GB research in the last century are depicted in Figure 1.

## 2 Glioblastoma Microenvironment

The microenvironment of GB is highly complex and dynamic with various cross-interacting components that contribute towards the tumor formation and invasion. Some of the components of the tumor microenvironment and their roles are described as under.

### 2.1 Blood-brain barrier

The blood-brain barrier (BBB) is a unique and specialized structure that regulates the flow of molecules between the blood and the brain (Martin et al., 2022a). The BBB serves as a physical barrier between the vascular system and the central nervous system. The BBB is primarily made up of endothelial cells, pericytes, and astrocytes which create a tight barrier along the walls of blood vessels, specifically limiting the substances that can enter the parenchyma (Martin et al., 2022b).

GB is characterized by a high degree of vascularity, atypical structure, and decreased structural integrity. This leads to leakiness causing high interstitial fluid pressure, severe ischemia and necrosis, and edema (Rehman et al., 2018). GB tumor cells produce a variety of factors, including VEGF and matrix metalloproteinases (MMPs), which degrade tight junction proteins and basement membrane components, resulting in greater BBB permeability. VEGF is a powerful angiogenic factor that supports the formation of new blood vessels in the tumor microenvironment while also increasing BBB permeability by downregulating tight junction proteins and raising endothelial cell fenestration. MMPs are enzymes that help tumor cells invasion and angiogenesis by eroding the extracellular matrix and basement membrane, as well as disturbing the BBB through tight junction protein degradation (Gregoriadis, 2008; Daneman and Prat, 2015).

BBB is critical for shielding the brain from toxins and viruses while allowing nutrient flow and gaseous exchange. Increased permeability triggers inflammatory responses, fostering chronic inflammation that hampers anti-tumor immunity. Therapeutic delivery is also affected, challenging treatments targeting the immune response. BBB dysfunction contributes to tumor-induced immune suppression, allowing the influx of immunosuppressive factors. Lastly, compromised immune surveillance results from the BBB’s failure, enabling tumor cells to evade detection and advance disease progression (Jain et al., 2007; Gomez-Zepeda et al., 2019). Increased BBB porosity allows immune cells and carcinogenic chemicals into the tumor microenvironment, encouraging tumor development and spread. Besides, it also limits therapeutic drug transport to the brain, reducing their effectiveness, and can contribute to cerebral edema, a frequent complication in GB (Louveau et al., 2015).

The increase in BBB permeability due to VEGF, MMPs and other by products formed during GB leads to remodeling of the BBB. Destruction of BBB leads to passage of large molecular weight substances into the extracellular fluid leading to passive diffusion of water resulting in cerebral edema. Progressive growth of tumor is often accompanied by overexpression of VEGF leading to neovascularization which further accelerates development of glioma. However, due to abnormal anatomy of new vascular tissue, the BBB permeability increases. The remodeled blood-brain tumor barrier (BTB) hinders the transport of therapeutic drugs due to the overexpression of extraneous (efflux) transporters on glioma cells. These efflux transporters belonging to the family of ABC transporters such as P-glycoprotein (P-GP), multidrug resistance associated protein (MRP), breast cancer resistance protein (BCRP) inhibiting the penetration of numerous therapeutic drugs by actively pumping these substrates out of the cells (Agarwal et al., 2011) resulting in poor efficacy of commonly used antitumor drugs against glioma (Parrish et al., 2015).

Development of new strategies to maintain the integrity of the BBB in GB, therefore, offers tremendous potential for improving patient outcomes. Although a spectrum of methodologies has been devised to navigate the complexities imposed by the blood-brain barrier, which includes direct drug delivery via intrathecal or intranasal routes, chemical modifications that target drugs or components of the BBB, suppression of efflux pumps, and physical disruption through techniques like radiofrequency electromagnetic radiation (EMP), laser-induced thermal therapy (LITT), focused ultrasounds (FUS) in combination with microbubbles, and convection-enhanced delivery (CED), but it frequently fails to reach therapeutic amounts in tumor in GB cells. (Wen et al., 2017; Quintero-Fabián et al., 2019; Mo et al., 2021).

### 2.2 The neuro-immune system in glioblastoma microenvironment

**FIGURE 2:**
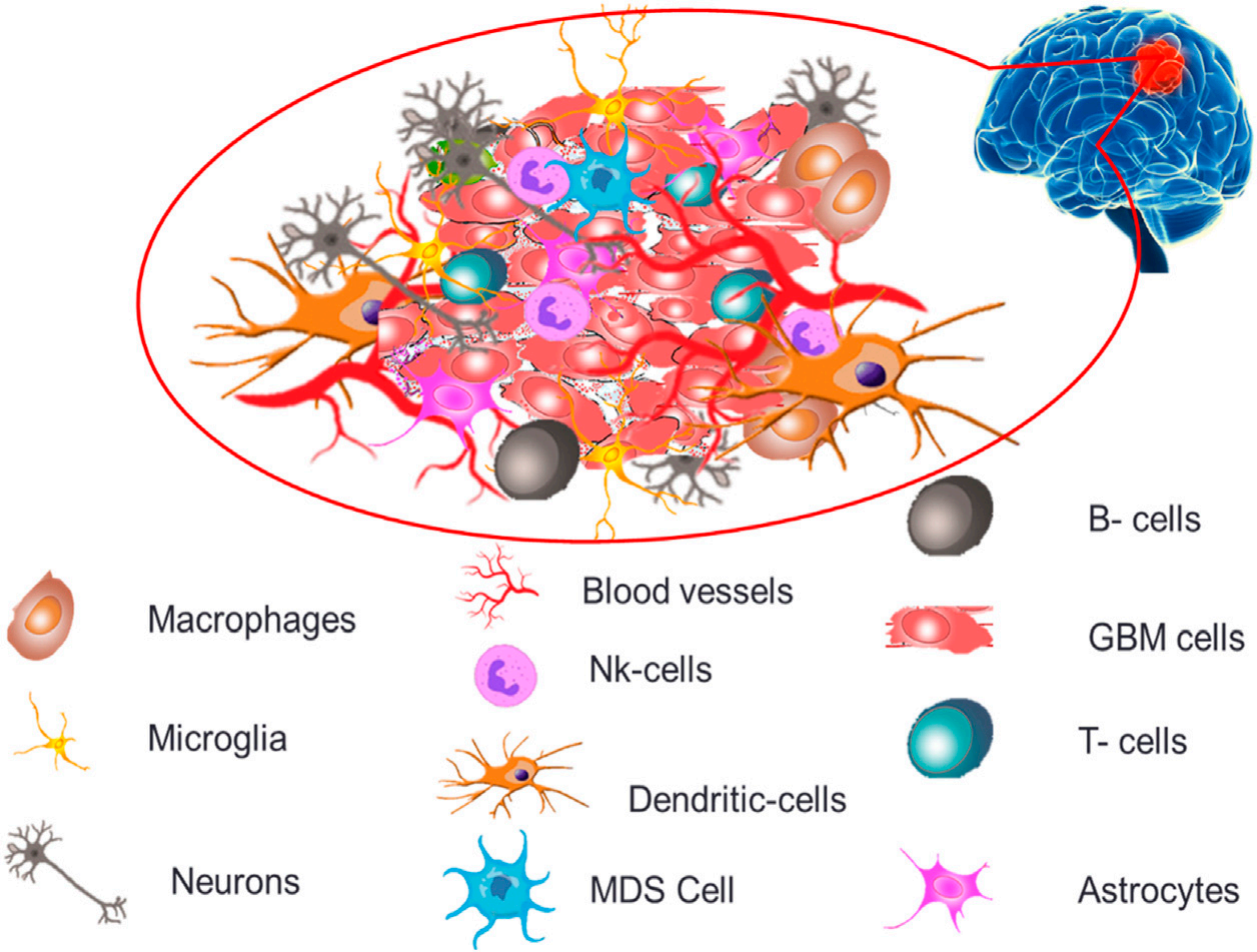
Diagrammatic illustration of the components of the GB microenvironment.

The permeability and cellular makeup of immune cells within the tumor microenvironment of GB are distinct, creating an extremely immunosuppressive microenvironment profile causing severe challenges for treatments and therapeutics (Zou et al., 2022). GB tumor microenvironment, as depicted in Figure 2 demonstrates both immune components (microglia, macrophages, T cells, B cells, NK cells, dendritic cells and myeloid-derived suppressor cells) and non-immune components (extracellular matrix, endothelial cells, astrocytes and neurons).

#### 2.2.1 Macrophages

Macrophages, also known as tumor-associated macrophages (TAMs), are one of the most common immune cells in the GB tumor microenvironment. Macrophages have been demonstrated to have both pro- and anti-tumor effects in cancer, depending on their polarization state and the tumor microenvironment (Liu et al., 2017). TAMs are drawn to the cancer site by chemokines like C-C motif chemokine ligand 2 (CCL2), and aid tumor development and invasion by secreting cytokines and growth factors including interleukin-6 (IL-6), TGF-β, and VEGF. TAMs also promote tumor growth by inhibiting anti-tumor immune responses and increasing angiogenesis. TAMs with M2-like characteristics produce markers such as Cluster of Differentiation 206 (CD206) and arginase 1 (ARG1) (Lamszus et al., 2003).

The colony-stimulating factor-1 receptor (CSF-1R) is a tyrosine kinase receptor expressed on macrophages that controls GB-associated macrophage survival, proliferation, differentiation, and polarization. CSF-1R interacts to its ligands (IL-34 and CSF-1), resulting in activation of PI3K, ERK, NFκB and Src mediated macrophage survival and proliferation pathways. Targeting CSF-1R can reduce GB-associated macrophages in the tumor microenvironment and promote GB-associated macrophage repolarization, limiting tumor development, avoiding the recurrence of glioblastoma and promoting cytotoxic T-cell activation (Vegliante et al., 2022).

Several approaches have been proposed to target TAMs in GB, including the use of small molecule inhibitors, monoclonal antibodies, and cell-based therapies. Targeting CCL2 or its receptor CCR2 has been proven in experimental GB models to reduce TAM infiltration and enhance GB cell mortality. The use of chimeric antigen receptor T-cells (CAR-T) that target TAM-specific markers such as CD163 is also being investigated as a viable treatment for GB (Lamszus et al., 2003). TAMs in GB are known to promote angiogenesis as well as tumor growth and invasion. TAMs produce pro-angiogenic factors such as VEGF, which promote blood vessel growth and proliferation in the cancer microenvironment (Vegliante et al., 2022). This procedure is crucial for the tumor development and progression of GB by catering to blood flow requirements for absorbing nutrients and oxygen. The use of chimeric antigen receptor CAR-T cells that target TAM-specific markers such as CD163 is being investigated as a potential therapy for GB (Chen et al., 2017).

According to recent studies, GB has at least two distinct macrophage populations: one that promotes tumor development and another that suppresses tumor growth (Achkova and Maher, 2016). Understanding the diversity of macrophages in the GB microenvironment may lead to the development of targeted therapies.

#### 2.2.2 Microglia

Microglia are brain immune cells that have been found to have an important role in the microenvironment of GB tumors. Microglia, in particular, are among the most abundant immune cells in the GB environment and are known as tumor-associated microglia. Tumor-associated microglia are drawn to the cancer site by chemokines such as CCL2, and help the tumor to expand and invade by secreting cytokines and growth factors such as IL-6, TGF-β, and VEGF. Tumor-associated microglia in GB are pro-tumor M2-like, as evidenced by the overexpression of markers such as CD206 and ARG1. These M2-like tumor-associated microglia have been demonstrated to assist tumor growth and invasion by inhibiting anti-tumor immune responses and increasing angiogenesis (Kioi et al., 2010; Zhou et al., 2015; Achkova and Maher, 2016).

Furthermore, tumor-associated microglia play an important role in GB treatment resistance, including radiation and chemotherapy. Tumor-associated microglia, in particular, can protect cancer cells from radiation-induced apoptosis by secreting cytokines like IL-6 and TNF-β, as well as increasing chemotherapy tolerance by upregulating drug efflux pumps (Philipsen et al., 2023).

Targeting microglia in GB has been proposed using small chemical inhibitors, monoclonal antibodies, and cell-based therapies. Targeting CCL2 or its receptor CCR2 in preclinical GB models, for example, has been shown to reduce TAM infiltration and boost survival (Mantovani et al., 2004; Wang et al., 2013).

#### 2.2.3 T cells

T cells are essential adaptive immune response mediators and play critical roles in anti-tumor immunity. T lymphocytes are present in the tumor microenvironment in GB. However, their activities are frequently compromised by a variety of mechanisms (Li et al., 2011) such as tumor-induced T cell fatigue, immunological checkpoint activation, and regulatory T cell (Treg) invasion. (Fecci et al., 2006). Tumor cells additionally release immunosuppressive cytokines such as transforming growth factor-beta (TGF-β), which inhibits T cell activity (Hussain et al., 2006). Depending on the subtype and functional state of the T cells, as well as the cytokine milieu inside the tumor microenvironment, infiltrating T cells can have both pro-tumorigenic and anti-tumorigenic effects (Wainwright et al., 2012).

Regulatory T cells (Tregs) Tregs, for example, have been demonstrated to promote glioblastoma development by inhibiting the anti-tumor immune response and establishing an immunosuppressive milieu (Berghoff et al., 2017). Effector T cells, on the other hand, have the ability to recognize and eliminate tumor cells but are frequently functionally impaired within the glioblastoma microenvironment due to factors such as the upregulation of immune checkpoint molecules and the accumulation of immunosuppressive cells (Alban, 2018).

#### 2.2.4 B cells

B cells are immune cells that generate antibodies and play an important part in the adaptive immune response. In the GB microenvironment, B cells can induce immune suppression and angiogenesis. They can generate cytokines including interleukin-10 (IL-10) and transforming growth factor-beta (TGF-β), which suppress the activity of immune cells like T-cells and NK cells while promoting GB development and invasion (Garg et al., 2017; Jiao et al., 2017). Furthermore, B cells can secrete pro-angiogenic factors such as VEGF, CXC chemokine ligand 12 (CXCL12), and CXC chemokine ligand 13 (CXCL13), which stimulate the formation of new blood vessels, provide nutrients and oxygen to the tumor, and promote GB growth and invasion (Shonka et al., 2013).

B cells also play a role in GB therapeutic response by influencing responsiveness to immune checkpoint inhibitors (ICIs). B lymphocytes can express immunological checkpoints including programmed death-ligand 1 (PD-L1), which interacts with the T cell immune checkpoint programmed cell death protein 1 (PD-1) to cause T cell depletion and immune evasion by GB cells (Pellegatta et al., 2015). Several approaches, including the use of monoclonal antibodies targeting B cells and B cell-derived cytokines, as well as B cell function modulation have been proposed to target B cells in the GB microenvironment. Anti-CD20 monoclonal antibodies, such as rituximab, can reduce B cells and increase survival in GB patients. In addition, inhibitors of B cell-derived cytokines such as IL-10 and TGF-β may be a potential strategy for GB treatment (Cai et al., 2015).

B cells play a role in immune escape and have a direct impact on GB development and progression. B cells isolated from GB patient samples have been shown to promote tumor cell proliferation and migration through the secretion of growth factors such as insulin-like growth factor-1 (IGF-1) and hepatocyte growth factor (HGF). They can also influence the immunological milieu of GB by secreting chemokines such as CXCL13, which is produced mostly by B cells in GB patient samples and is related to enhanced T cell infiltration and improved patient survival (Khan et al., 2020).

Anti-CD20 antibodies like as rituximab have been studied in clinical trials for the reduction of B cells in GB. Combination of Anti-CD20 antibodies with traditional chemotherapy medicines such as temozolomide has increased progression-free survival. Rituximab has also been demonstrated to diminish B cell infiltration in the GB microenvironment and increase immunotherapy effectiveness (Wintterle et al., 2003).

#### 2.2.5 Natural -killer cell

NK cells are innate immune cells that help the body defend against viruses and tumors. NK cells are present in the tumor microenvironment in GB but are frequently functionally compromised (Vivier et al., 2008). NK cells have been demonstrated in studies to infiltrate glioblastomas and may contribute to tumor management via a variety of methods, including the direct killing of tumor cells and activation of other immune cells (Yang et al., 2010). However, NK cells, like other immune cells, can be suppressed within the glioblastoma microenvironment by factors such as immune checkpoint molecule upregulation and immunosuppressive cell accumulation (Hasmim et al., 2015). Tumor cells can cause NK cell dysfunction through a variety of methods, including activating ligand downregulation and inhibitory ligand overexpression (Fujita et al., 2011). Immunosuppressive substances in the tumor microenvironment, such as TGF-β and interleukin-10 (IL-10) (Ho and Ho, 2021), can also limit NK cell activity.

#### 2.2.6 Dendritic cells

Dendritic cells (DCs) are important components of the immune response and have been linked to glioblastoma (GB) tumor microenvironment. Recent research has shown that DCs can interact with other immune cells in the GB microenvironment, boosting the growth of regulatory T cells (Tregs) through TGF-mediated signaling (Carmeliet and Jain, 2000). DCs release VEGF-A, which promotes tumor development and angiogenesis in GB (Plate et al., 1992b). The presence of DCs in the GB microenvironment implies requires a better understanding into their interactions with other immune cells, that may lead to better treatment methods. Boosting anti-tumor immunity of DCs while suppressing their pro-tumor actions can be an effective strategy for GB therapy.

#### 2.2.7 Myeloid-derived suppressor cells

MDSCs, a diverse population of immature myeloid cells that limit the function of immune effector cells. Several studies have shown that MDSCs promote tumor growth and invasion via a variety of mechanisms, including cytokine and growth factor secretion, immune effector cell suppression, and angiogenesis stimulation. MDSCs secrete IL-6 by activating the signal transducer and activator of transcription 3 (STAT3) signaling pathway, promoting tumor growth and invasion. MDSCs also inhibit NK cell activity by upregulating PD-L1 expression (Eichler et al., 2011).

Depleting MDSCs in the tumor microenvironment can increase the activity of immune effector cells while decreasing tumor development and invasion. In a mouse model of GB, all-trans retinoic acid (ATRA)-mediated MDSC reduction enhanced T cell infiltration and decreased tumor development (Sun et al., 2021). Similarly, targeting S100 Calcium Binding Protein A9 (S100A9), which is expressed by MDSCs, inhibited tumor development and invasion in a mouse model of GB (Hynes, 2002).

### 2.3 Non immune cells in glioblastoma micro-environment

#### 2.3.1 Endothelial cells

Endothelial cells present in the tumor microenvironment play an important role in supporting glioblastoma development and invasion (Reardon et al., 2017). Endothelial cells are important for angiogenesis, which is the development of new blood vessels from pre-existing ones and is a characteristic of glioblastoma growth. These cells have high quantities of pro-angiogenic factors including VEGF, which promote blood vessel creation as well as glioblastoma cell survival and invasion (Silva et al., 2008). Endothelial cells can also influence the immune response by secreting immunosuppressive factors like TGF-β and expressing immunological checkpoint molecules like PD-L1 that decrease T cell activation and proliferation. Endothelial cells can also modify the extracellular matrix (ECM) of glioblastoma tumors, allowing glioblastoma cells to migrate and invade (Tawi et al., 2021).

Several strategies have been developed to target endothelial cells in glioblastoma therapy. One such strategy is to use monoclonal antibodies or small molecule inhibitors to limit VEGF signaling and hence reduce angiogenesis. Another strategy is to target endothelial cell survival and migratory pathways such as PI3K/Akt and mTOR (Charles et al., 2012). However, these approaches have limitations and can interfere with normal endothelial cell functions, resulting in negative outcomes (Liang et al., 2014). Immunotherapy is another intriguing strategy that targets the immune regulatory activities of endothelial cells. Anti-PD-L1 antibodies have shown potential therapeutic effects in glioblastoma tumors by increasing T-cell infiltration and activation. In preclinical investigations, combining anti-PD-L1 treatment with other immune checkpoint inhibitors, such as anti-cytotoxic T-lymphocyte–associated antigen 4 (anti-CTLA-4), yielded encouraging outcomes (Cekanaviciute et al., 2014). However, more research is needed to develop safe and effective therapeutic strategies for glioblastoma tumors that target endothelial cells.

#### 2.3.2 Astrocytes

Astrocytes, the most common glial cells in the central nervous system, have been revealed to play an important role in glioblastoma tumor microenvironment. Glioblastoma tumor-induced astrocyte activation can increase tumor growth. Activated astrocytes release cytokines and growth factors that promote glioblastoma cell proliferation and invasion. Astrocyte activation has also been linked to a poor prognosis in glioblastoma patients (Olea-Flores et al., 2020). Astrocytes can produce immunomodulatory substances such transforming growth factor- (TGF-β) and interleukin-10 (IL-10), which dampen the immune response to glioblastoma cells. Furthermore, astrocytes can inhibit the activity of immune cells involved in anti-tumor immunity, such as T-cells and NK-cells (Farin et al., 2006).

Astrocytes, in addition to immunological regulation, can enhance angiogenesis in the glioblastoma microenvironment. Astrocytes also have the ability to produce vascular endothelial growth factor (VEGF), a critical regulator of angiogenesis, and express integrins as well as extracellular matrix proteins that aid in the formation and stabilization of blood vessels (Nagy et al., 2010). Astrocytes have also been found to contribute to drug resistance in glioblastoma tumors by secreting factors that protect glioblastoma cells from chemotherapy. Additionally, astrocytes can form a physical barrier between glioblastoma cells and chemotherapy agents, preventing them from reaching the tumor cells. They can also activate signaling pathways that promote drug resistance in glioblastoma cells (Zhao et al., 2008). Understanding the role of astrocytes in glioblastoma tumor microenvironment is essential for the development of effective treatments for this devastating disease.

#### 2.3.3 Neurons

In recent research, neurons in the GB microenvironment have been identified as a potential contributor to tumor formation. Glutamate and substance P, for example, have been shown to promote GB cell motility and invasion. It has been demonstrated that the synthesis of chemicals like brain-derived neurotrophic factor (BDNF) by neurons can promote cancer growth by boosting angiogenesis (Venkatesh et al., 2017).

Neurons in the GB microenvironment are not passive bystanders; rather, they engage in active interactions with glioblastoma cells. Glioblastoma cells on the other hand promote the formation of synapses between neurons, resulting in enhanced neuronal activity (Venneti et al., 2015). Neurons, have been shown to alter glioblastoma cell behaviour through the synthesis of BDNF and neuregulin-1 (NRG1) (Venkataramani et al., 2019). Recent research has shown that glioblastoma cells can form direct synapses with neurons, allowing impulses to be transmitted between the 2 cell types (Lu et al., 2012).

The interaction of neurons and glioma cells involves complex signaling pathways (Venkatesh et al., 2019). The binding of BDNF to its receptor TrkB on glioblastoma cells, for example, has been shown to promote tumor development and invasion by activating many signaling pathways such as PI3K/AKT, MEK/ERK, and PLC. Similarly, binding of NRG1 to its receptor ErbB3 on glioblastoma cells has been shown to increase cancer cell survival and invasion via the PI3K/AKT and MEK/ERK pathways (Kermani et al., 2005).

The discovery of neurons’ participation in the GB microenvironment has significant implications for GB treatment (Zhang et al., 2008). In preclinical studies, blocking BDNF signaling was shown to reduce GB development and invasion. Inhibiting NRG1 signaling also improves glioblastoma cell susceptibility to radiation treatment (Zhang et al., 2014). Targeting neuronal signaling pathways, could therefore emerge as a novel therapeutic strategy for GB.

## 3 Single-cell sequences in GBM

Single-cell sequencing has revolutionized our understanding of the complex genetic heterogeneity driving tumor growth by providing a means of analyzing genomes, transcriptomes, and epigenomes at the individual cell level. It reveals a level of complexity that was previously unattainable by identifying cellular heterogeneity, which is frequently hidden in hybrid sample sequencing carried out conventionally (Risau, 1997). Concurrently, the Tumor Microenvironment (TME) becomes apparent as a crucial element in the emergence, progression, and reaction to treatment of tumors. A paradigm shift in TME analysis has been brought about by the application of single-cell sequencing, which has revealed the immune cells' differentiation pathways and heterogeneity as well as the pan-cancer immune microenvironment blueprint, providing prognostic value for tumor prognosis. This collaboration offers a singular chance to analyze the molecular processes driving the genesis and spread of tumors (Hanahan and Folkman, 1996). Advances in TME analysis and single-cell sequencing have shown great promise recently, with applications ranging from clinical translation to cancer research. Through single-cell transcriptome sequencing and spatial transcriptome combination, the previously underappreciated presence of tumor-associated fibroblasts (CAFs) in glioblastoma (GBM) has been revealed, challenging the presumption and advancing our knowledge of GBM (Ferrara and Kerbel, 2005). Understanding the fibrocytes that promote tumor growth improves our understanding of GBM and opens the door to more intelligent treatment approaches. Given that tumor cells can adapt to changes in their environment brought about by drugs, it is critical to comprehend the unique differentiation processes that these cells go through to develop targeted therapies that are effective (Navin et al., 2011).

## 4 Mechanisms pertaining to progression of GB

### 4.1 Angiogenesis in GB

**FIGURE 3:**
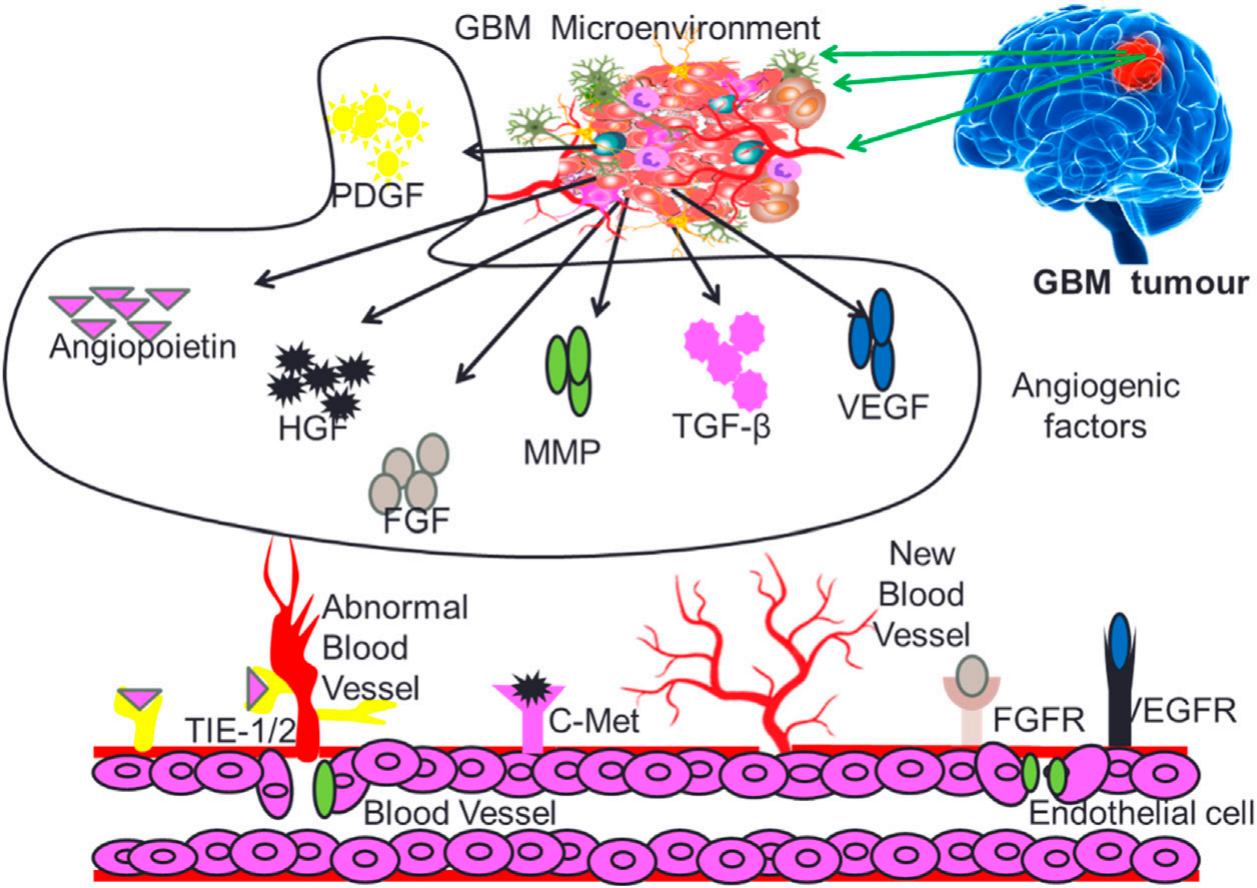
Different types of growth factors supporting GB tumor growth and proliferation.

Angiogenesis is a critical process for the development and maintenance of blood vessels in various tissues, and its dysregulation has been implicated in the progression of multiple diseases, including cancer and inflammation (Puram et al., 2017; Jain et al., 2023). The angiogenesis induced neovascularization in tumors is critical for providing required nutrients and oxygen to fast growing GB cells. Angiogenesis in the GB micro-environment is modulated by several growth factors that include vascular endothelial growth factor (VEGF), hepatocyte growth factor (HGF), Fibroblast growth factor (FGF), Platelet-derived growth factor (PDGF), Transforming growth factors (TGF-β), Matrix metalloproteinase (MMPs) and Angiopoietins (Angs) as depicted in Figure 3.

#### 4.1.1 Vascular endothelial growth factors

Vascular endothelial growth factors (VEGFs) and receptors (VEGFRs) play critical roles in vasculogenesis and angiogenesis (Quail and Joyce, 2017). Endothelial cell proliferation and survival are largely driven by the ERK, neuropilin-1 (NRP1), and PI3K/Akt pathways, which are triggered by a variety of signaling intermediates (Takahashi et al., 2001). Endothelial cell migration is regulated by several mechanisms that frequently converge on PI3K stimulation, resulting in the activation of Rho family G proteins. VEGF can also attach to NRP1 on endothelial cells, boosting VEGF binding to VEGFR2 and magnifying its pro-angiogenic effects (Garrido-Urbani et al., 2009). MMPs are upregulated by VEGFA predominantly through Akt-dependent activation of -catenin and nuclear factor kappa B (NFκ-B) and ROS-mediated mechanisms (Van Hinsbergh and Koolwijk, 2008).

Following the discovery of VEGF as a key regulator of pathologic angiogenesis and tumor growth in glioblastoma, VEGF and VEGFR inhibitors have been developed (Gerstner et al., 2009). The first anti-angiogenic medication approved for cancer therapy was bevacizumab, a human monoclonal antibody that precisely recognizes and inhibits VEGF-A (Vredenburgh et al., 2007). However, resistance mechanisms such as the activation of alternative angiogenic pathways and the recruitment of pro-angiogenic immune cells have limited anti-angiogenic therapy’s clinical effectiveness in glioblastoma (Gilbert et al., 2014).

#### 4.1.2 Fibroblast growth factors

FGFs are a class of growth factors that govern angiogenesis and have been linked to the development of GB. FGFs bind to four tyrosine kinase receptors (FGFR1-4) on the cell surface, activating a variety of signaling pathways such as PI3K, PLC, mitogen-activated protein kinases (MAPK), signal transducers and activators of transcription (STATs), and protein kinase C (PKC). These mechanisms increase cell proliferation, survival, and migration, which are required for tumor angiogenesis (Hadari et al., 2001). FGF expression is elevated in glioblastoma and is associated with tumor grade and patient prognosis. FGFs enhance angiogenesis by boosting endothelial cell proliferation and migration (Udayakumar et al., 2002), triggering the production of pro-angiogenic molecules such as VEGF, and affecting extracellular matrix remodeling during angiogenesis (Takahashi et al., 1992). Because of FGF’s crucial involvement in glioblastoma tumor angiogenesis, treatments targeting FGF signaling pathways, such as FGFR inhibitors and combination therapies targeting FGF and other pro-angiogenic pathways such as VEGF, have been developed (Shah et al., 2008; Koch et al., 2012).

#### 4.1.3 Platelet-derived growth factor

Platelet-derived growth factor (PDGF) family members include PDGF-AA, AB, BB, CC, and DD, which activate intracellular signaling pathways such as Ras-MAPK, PI3K, and PLCg (Vredenburgh et al., 2007) to kick-start the cell cycle, DNA synthesis, mitosis, cell migration, and chemotaxis (Galanis et al., 2018). GB is characterized by elevated PDGF and PDGFR expression. Preclinical investigation in rats and marmosets revealed that PDGF overexpression, particularly PDGFB and PDGFA play an important role in the genesis and progression of GB (Saha and Giri, 2017). Furthermore, it has been demonstrated that low molecular weight PDGF receptor kinase inhibitors effectively reverse the transformed phenotype and inhibit tumor growth *in vivo*. In GB, PDGF/PDGFR targeting has shown promising therapeutic outcomes (Xu et al., 2015). PDGF genes express differently in glial cells and endothelial cells in GB micro-environment, while PDGFR is preferentially expressed in GB stem cells, encouraging their self-renewal and invasion (Ozawa et al., 2014). Endo-MT and VEGFR-2 down-expression are regulated by the PDGF/NF-B/Snail axis in GB, resulting in resistance to anti-angiogenic treatment. Inhibiting PDGF signaling improves the sensitivity of anti-VEGF/VEGFR therapy in GB, allowing for the creation of more effective treatment regimens (Uhrbom et al., 2004). PDGF-D is a potent growth factor for human GB cells, and HIF1 plays an important role in the constitutive activation of the AKT signaling pathway in GB by regulating the expression of PDGF-D and its receptor, PDGFR. Inhibition of HIF1α disrupts the HIF1α-PDGF-D-PDGFRα feedforward axis, leading to the abolishment of AKT pathway activation and a potentially effective therapeutic approach for GB treatment (Liu et al., 2018).

#### 4.1.4 Hepatocyte growth factor

Hepatocyte growth factor (HGF), also known as scatter factor (SF), is a heparin-binding mesenchyme-derived cytokine that has been linked to glioblastoma multiforme (GB) angiogenic process. HGF/SF has been established in recent investigations to play an important role in GB tumor angiogenesis by boosting endothelial cell proliferation, migration, and survival (Peng et al., 2021).

HGF/SF is a strong mitogen that promotes endothelial cell growth. The binding of HGF/SF to its receptor, c-MET, stimulates multiple downstream signaling pathways that promote cell proliferation, including the PI3K-AKT, RAS-ERK, and JAK-STAT pathways. HGF/SF has been demonstrated to increase endothelial cell proliferation in GB by activating the PI3K-AKT and RAS-ERK pathways (Trusolino and Comoglio, 2002).

The hepatocyte growth factor/scatter factor (HGF/SF) has been linked to the control of angiogenesis through the overexpression of VEGF, a powerful pro-angiogenic factor, and the reduction of thrombospondin 1 (TSP-1), an endogenous regulator of angiogenesis. This dual method of action shows that HGF/SF is important in balancing pro- and anti-angiogenic factors in the tumor microenvironment (Olmez et al., 2018).

#### 4.1.5 Angiopoietins (Angs)

Angiopoietins (Angs) are a protein family that binds to the Tie-2 receptor and play an important role in GB angiogenesis. Angiopoietins are classified into four kinds, each with distinct and opposing actions on the vasculature (Knowles et al., 2009). Angiopoietin-1 (Ang-1) promotes endothelial cell survival, proliferation, migration, and tube formation by activating the Tie2 receptor tyrosine kinase and stabilizing blood vessels. It also stimulates the recruitment of pericytes and smooth muscle cells to the vessel wall, which helps to stabilize it. Ang-1 causes new vessel development with angiogenic activities independent of VEGF and stabilizes them via reciprocal interactions between the endothelium and the surrounding extracellular matrix (Suri et al., 1996a; Suri et al., 1996b).

Ang-1 and Ang-2 are key Ang family members that connect to the Tie-2 receptor on endothelial cells. Ang-1 promotes blood vessel stabilization, whereas Ang-2 promotes vascular remodeling by destabilizing blood vessels and inducing sprouting angiogenesis (Daly et al., 2006a). Ang 2 also stimulates downstream signaling pathways that induce angiogenesis, such as AKT and ERK (Ricci-Vitiani et al., 2010). The invasiveness of gliomas is linked to Ang-2 upregulation. Inhibiting Ang-2 expression can reduce glioma invasiveness and avoid an anti-VEGF therapeutic escape mechanism. Dual suppression of the VEGF and angiopoietin-TIE2 signaling pathways is thought to be a potential treatment option for recurrent GB (Kienast et al., 2010). The role of Ang-1 in GB angiogenesis on the other hand is controversial, with some studies showing that it can suppress GB angiogenesis by stabilizing blood vessels and reducing vessel permeability (Huang et al., 2010).

#### 4.1.6 Transforming growth factors (TGF-β)

The TGF- family of structurally similar polypeptides regulate several pro-tumorigenic processes, including proliferation, apoptosis, differentiation, epithelial-mesenchymal transition (EMT), and angiogenesis. TGF-β signaling can activate downstream effectors that govern cellular activities such as the canonical Smad pathway, which includes the Wnt, Notch, Hippo, Hedgehog, and JAK-STAT pathways, as well as non-Smad pathways such as PI3K-Akt, MAPK, and Rho GTPases (Akwii et al., 2019).

The key process of epithelial-mesenchymal transition (EMT) promotes the growth of GB. E2 and TGF-β have been found to suppress the expression of the estrogen receptor (ER) and Smad2/3. TGF-β causes Smad2 phosphorylation and subsequent nuclear translocation, which is blocked by E2. Both E2 and TGF- β have been discovered to elicit EMT-related cellular processes such as morphological alterations, actin filament reorganization, and mesenchymal marker expression (N-cadherin and vimentin). Interestingly, co-treatment with E2 and TGF- β has been shown to inhibit EMT activation (Daly et al., 2006b).

TGF- β is linked with a poor prognosis in GB and promotes the production of pro-angiogenic factors such as VEGF, FGF, and PDGF-β (Itoh et al., 2018). TGF-β1 enhances glioma-induced angiogenesis via the JNK pathway in zebrafish embryo/xenograft glioma models (Li and Flavell, 2008). Crosstalk between TGF-β and VEGF/PLGF signaling has been demonstrated to exhibit both pro- and anti-angiogenic actions in human brain-derived microvascular endothelial cells (hCMECs) and glioblastoma-derived endothelial cells (GMECs). TGF-β increases VEGF and PlGF mRNA and protein expression in glioma cells, resulting in pro-angiogenic effects. Exogenous TGF-β, on the other hand, inhibits endothelial characteristics and causes endothelial-mesenchymal transition (EndoMT) in hCMEC and GMEC (Liu et al., 2012).

#### 4.1.7 Matrix metalloproteinases

Matrix metallopeptidases (MMPs) are a class of structurally conserved zinc-dependent endopeptidases generated by tumor cells that play a crucial role in tumor development invasion, metastasis, and angiogenesis in the central nervous system through participating in the breakdown of extracellular matrix macromolecules such as angiogenesis and cytokine activation. MMPs stimulate endothelial cell invasion by destroying the vascular basement membrane and ECM (Hernández-Vega and Camacho-Arroyo, 2021). They also promote pericyte proliferation and activation, as well as their migration to newly formed vasculature, resulting in vascular stabilization (Chamberlain, 2013). MMP-9-mediated VEGF release from the matrix has been shown in multiple transgenic mice models that triggers angiogenic switching in pre-malignant tumors (Krishnan et al., 2015).

Guanylate-binding proteins 5 (GBP5) mediated activation of MMP3 plays an important role in the development and invasion of glioblastoma (GB). GBP5 has been demonstrated to activate the Src/ERK1/2 MAPK pathway in GB cells, resulting in the production of matrix metalloproteinase-3 (MMP3). According to research, MMP3 is overexpressed in gliomas and contributes to the aggressiveness of GB. As a result, the GBP5/Src/ERK1/2/MMP3 axis has been postulated as a possible therapeutic target for GB (Sternlicht and Werb, 2001; Bassiouni et al., 2021).

## 5 Therapeutic implications in GB

GB treatment is particularly challenging due to its intra-tumor heterogeneity wherein the cells have differences in characteristics at molecular levels which is further complicated by genetic and epigenetic changes that promote evasion of standard treatment procedures. Neoplastic cells demonstrate the quality to adjust to their surrounding environment and accordingly alter their microenvironment for their advantage. This complexity leads to the need for varying therapeutic strategies which can act together to provide a meaningful therapeutic response. GB patients are basically treated by surgical exclusion of the neoplastic lesion usually followed by chemotherapy and radiation therapy. With the advent of modern research, a number of new techniques have been put forward towards the treatment of GB patients (Pombo Antunes et al., 2020) There are new approaches to the problem of BBB blockage, but before they are used in clinical settings, their feasibility must be confirmed.

### 5.1 Adaptive T cells

The most common chemotherapeutic agent is temozolomide, a DNA methylator that causes DNA cross-linking. Adaptive T cells therapies which include IL13Rα2-CAR-T cells, IL13Rα2 is known to promote tumor regression in glioma and few other tumor models. With the increase in malignancy grade expression of IL13Rα2 is a prognostic indicator for poor patient survival (Chantrain et al., 2006).In recent times personalized cancer therapy is emerging as a preferred therapy for cancer treatment. One such method is treating cancer-bearing host with Cytokine-induced killer (CIK) immune cells which can direct an anticancer activity. Cytokine-induced killer (CIK) cells are major histocompatibility (MHC)-unrestricted cytotoxic lymphocytes that can be produced *in vitro* from peripheral blood mononuclear cells and can also be cultured with the addition of interferon (IFN)-γ, interleukin (IL)-2, and CD3 monoclonal antibody (CD3mAb). (Brown et al., 2013). The use of immunotherapy, to provide anti-tumor immune response is surely an area of active research towards the treatment of glioblastoma.

Promising survival advantages were reported in both *in vitro* and in xenograft models when CAR-T treatment targeting CD276 was used (Wang et al., 2014). A unique understanding of the combination of anti-CD276 and anti-angiogenesis revealed by using antibody–drug conjugates to ablate CD276+glioma cells concurrently impairing tumour vascular, which was reinforced by the confirmation that CD276 was positively correlated with VEGFA and MMP2. In patients with GBM, CAR-T cells that target EGFRvIII, HER2, and IL-13Rα2 have demonstrated a good safety profile and therapeutic effects.

### 5.2 Checkpoint Inhibitor

In the physiologic state, immune checkpoints play a major role in the sustainable balance of immune modulation in order to prevent autoimmune disorders. Immune checkpoints are the key regulators of the immune system that are expressed by T effector cells, antigen-presenting cells (APCs), and myeloid-derived cells in the normal immune system. When these checkpoints are engaged, they reduce immune activity by inducing T-cell anergy and apoptosis. Programmed death ligand 1 (PD-L1) mediated immune evasion plays a major role in neoplastic growth. Programmed death 1(PD1) and its ligand PDL1 regulate the proliferation and activation of T cells. Blockage of immune checkpoints with monoclonal antibodies has emerged as a promising new approach for the treatment of glial neoplasms. Previous studies on murine glioma models show improved survival with the use of checkpoint inhibitors that suggest immune checkpoint blockade can play an important role in treatment options for GB. (Berghoff et al., 2015; Nehama et al., 2019).

In a phase I trial, the co-inhibitory molecule T cell immunoglobulin and mucin domain-containing protein 3 (TIM-3), which is expressed on immune cells, is being investigated for its inhibition in relation to anti-PD1 medications for a variety of tumour types, including GB. Lymphocyte activation gene 3 (LAG-3) served as an early indicator of exhausted T cells, suggesting that early anti-LAG-3 medication therapy may be beneficial. A phase I trial is being conducted to examine the effects of anti-LAG-3 treatment alone or in conjunction with anti-PD1 treatment for patients with recurrent GBM (Reardon et al., 2021). AntiTIGIT drugs are direct inhibitors of T-cell proliferation and improve the anti-tumor immune response in many pre-clinical studies as a monotherapy or in combination with PD-1 and TIM-3 inhibitors. T cell immunoreceptor with Ig and ITIM domains (TIGIT) is an inhibitory receptor expressed on several types of lymphocytes. Anti-TIGIT treatment is reported to be currently in phase I clinical development for recurrent GBM in a multicenter trial in combination with antiPD-1 drugs (Lim et al., 2017).

Nivolumab is a fully human immunoglobulin G4 monoclonal antibody that targets the programmed death-1 (PD-1) immune checkpoint receptor (Zhang et al., 2018). Ipilimumab, a human IgG1 monoclonal antibody, is directed against CTLA-4, a critical immune checkpoint molecule known to be overexpressed on the membranes of Treg and downregulates the function of the effector cells. By increasing this inhibitory effect, ipilimumab stimulates the immune system and enhances its reaction against the tumor (Reardon et al., 2020). Pembrolizumab, which is a human anti-PD-1 antibody has shown antitumor activity with a favorable safety profile and has also been approved by the US Food and Drug Administration (FDA) as monotherapy across a number of tumor types (Youssef and Dietrich, 2020).

### 5.3 Angiogenesis inhibitors

It is now known that VEGF, also referred to as VEGF-A is an important regulator of angiogenesis. Human anti VEGF monoclonal antibody like (rhuMab VEGF; bevacizumab; Avastin) binds with VEGF with a very similar to that of the original antibody (Kd ∼0.5 nM) and thereby prevents its interaction with VEGF receptor tyrosine kinases VEGFR1 and VEGFR2 on the surface of endothelial cells. (Burns et al., 2016).

### 5.4 Vaccine approach

Dendritic cell (DC) vaccines are potentially effective in the treatment of GB, though their development is quite a challenging process requiring elaborate facilities to safely extract and pulse DCs with the tumor components. This limits the ability of DC vaccines to be rapidly scaled for broader utilization. Similar to DC vaccines, peptide vaccines are made from the tumor associated antigens. However, unlike DC vaccines that are personalized therapies, peptide vaccines are produced centrally. Thus, peptide vaccines can be distributed to different medical facilities more quickly, which makes them a viable strategy for multicenter glioma immune treatment trials.

A well-known target for peptide vaccines is the epidermal growth factor receptor (EGFR), which is a receptor tyrosine kinase that is highly expressed in glioblastoma. In GB, the most frequent mutation of EGFR is the EGFRvIII truncated variant that aids signaling independent of ligands. Constitutively active kinase activity and the existence of distinct amino acid sequences at newly formed mutation-induced junctions are the outcomes of an in-frame deletion of EGFR exons 2 through 7. EGFRvIII therefore promotes glioma tumors. This makes EGFRvIII an important target for immune therapy, as it is a tumor-specific target driver of the malignancy and is expressed on the cell surface. (Ferrara et al., 2004).

Rindopepimut is a 14-mer peptide conjugated to the immunogenic carrier protein keyhole limpet hemocyanin (KLH) that spans the mutation site of EGFRvIII (PEPvIII: NH2-Leu-Glu-Glu-Lys-Lys-Gly-Asn-Tyr-Val-Val-Thr-Asp-His-Cyt-COOH) (Garima et al., 2022). Also known as CDX-110, it targets the EGFR deletion mutation EGFRvIII, consisting of an EGFRvIII-specific peptide conjugated to keyhole limpet hemocyanin. Rindopepimut is under phase 3 clinical trials in glioblastoma patients (Swartz et al., 2014). The vaccine is injected intradermally leading to antigen recognition by antigen-presenting cells (APCS) and its presentation to T-cells cytotoxic and T-lymphocyte (CTL) T-cells which in turn activate B-cells that produce antibodies against EGFRvIII in the tumor. CTLs have the ability to cross the blood-brain barrier and target GB cells with EGFRvIII mutation on the surface. This activation of T-cells and CTLs leads to an anti-tumor response (Weller et al., 2017).

Tumour necrosis factor superfamily receptor abundant on T cells and glucosecorticoid-induced TNFR-related protein (GITR) has showed potential as an immunotherapy target. An agonistic antibody against GITR has been reported to inhibit Tregs in a number of models and has been addressed in glioma. Granzyme B (GrB) expression by Tregs was significantly reduced by peripheral treatment, whereas GrB expression by Tregs was also selectively depleted by intratumoral treatment, primarily by FcγR-mediated destruction. Anti-GITR treatment results in the enhanced survival and functionality of dendritic cells (DCs). (Guo et al., 2015).

### 5.5 Signaling pathway targets

Genomic analysis of glioblastoma revealed several signaling pathways and gene alterations that are critical for the development and progression of GB. The conversion of sphingomyelin to ceramide which is regulated by the enzyme sphingomyelin phosphodiesterase 1 (SMPD1) is one such pathway with SMPD1 being a potential drug target for GB. The highly brain-penetrant antidepressant fluoxetine has an inhibitory effect on SMPD1 activity, thereby killing GBMs by inhibiting the epidermal growth factor receptor (EGFR) signaling and activation of lysosomal stress mechanisms. (Miska et al., 2016; Lynes et al., 2018).

Nonsteroidal anti-inflammatory drugs (NSAIDs) act as a cell proliferation inhibitor by suppressing Wnt/b-catenin/Tcf signaling in many human malignancies. Studies have revealed that NSAIDs diclofenac and celecoxib are potential therapeutic agents that target glioblastoma cells by downregulating the activation of Wnt/b-catenin/Tcf signaling (Bi et al., 2021; Ulrich et al., 2006; mT hun et al., 2002).

Tumor cells induce vasogenic edema by releasing VEGF which induces increased vascular permeability in the tumor microenvironment. Dexamethasone, a synthetic glucocorticoid, counteracts this process by acting on glucocorticoid receptors, thereby decreasing both VEGF expression by tumor cells and VEGF sensitivity of the endothelial target cells (Sareddy et al., 2013).

Micheliolide (MCL) derivative 9-oxomicheliolide, has a strong anticancer activity towards GB in mice, with its rate of tumor inhibition comparable to clinical drug temozolomide. The mode of action of 9-oxomicheliolide is apparently through inhibitory effects on NF-κB and STAT3 signaling pathways as well as induction of cell apoptosis (Swildens et al., 2022).

The epidermal growth factor receptor-1 (EGFR) is a protein tyrosine kinase that can be activated by both epidermal growth factor (EGF) and tumor growth factor-α (TGF-α). Reports suggest that there is an upregulation of EGFR receptors in glioblastoma patients. (Zeng et al., 2021). Erlotinib is an orally bioavailable reversible competitive inhibitor of the adenosine triphosphate region of the EGFR tyrosine kinase domain. The FDA has approved erlotinib for use as a second and third-line treatment for non-small cell (NSCL) cancer (Wong et al., 1987).

Integrins are transmembrane αβ heterodimers; in humans, there are at least 18 α and 8 β subunits identified, resulting in 24 heterodimers. Members of this family are found in chicken, mammals, and zebrafish, as well as lower eukaryotes, including sponges, the nematode *Caenorhabditis elegans* (two α and one β subunits, which generate two integrins) and the fruitfly *Drosophila melanogaster* (five α and one β, which generate five integrins) (De Groot et al., 2008).

In a phase I trial, individuals with reccurent GBM showed excellent tolerance to cediranib plus celenigitide, a selective integrin inhibitor that targets αvβ3 and αvβ. Cilengitide (CGT) has a moderate efficacy in a phase II study and can bind with avβ3 and αvβ5 to be transported and stored in reccurentGBM cells (Takada et al., 2007). It has been reported that intravenously injected nanoparticles enhance drug penetration into the central nervous system by raising the permeability of the blood-brain barrier. In addition to preventing medication exposure to off-target tissues, encapsulating CGT in a well-designed nanocarrier can postpone drug clearance and enable prolonged or triggered release. This is especially important for CGT therapy since it can prolong its circulation duration, achieve more favorable pharmacokinetics, and lessen its non-specific effects on healthy cells, making the possibility of safer and more effective cancer treatment possible (Yang et al., 2022).

### 5.6 Oncolytic virus

Because of their modest pathogenic effect, oncolytic viruses (OVs) have antineoplastic roles by selectively infecting and killing cancer cells while sparing healthy ones. Furthermore, OVs may be able to change the immunosuppressive Tumor Microenvironment (TME) to an immunocompetent one. In fact, OV has the ability to carry a transgene and releases tumour antigens (Zhao et al., 2016).

DNX-2401 is a replicative engineered oncolytic adenovirus that consists of two stable genetic changes in the adenovirus genome for selectively and efficiently infecting and replicateing retinoblastoma pathway deficient such as tumor cells (Ma et al., 2023). Preclinical studies on DNX-2401 has demonstrated to be effective against glioma xenograft mouse models received through intratumoral injections of the virus by direct oncolysis in addition to eliciting antitumor immune responses. Phase I trials were carried in GB patients. For patients with recurrent GB or gliosarcoma, DNX2401 in combination with pembrolizumab, an anti-PD-1 antibody, is being tested in a phase II trial (Jiang et al., 2014).

Recombinant Lerapolturev (PVSRIPO), a rhinovirus chimaera derived from poliovirus (PV), is a new intratumoral immunotherapy that is non-neurovirulent. According to trial data in patients with rGBM, PVSRIPO monotherapy has a higher long-term survival rate. PVSRIPO targets CD155, the PV receptor, which is expressed in APC and solid tumours. In TME APC, PVSRIPO infection causes non-lethal persistent infection but inflammatory-mediated tumour cell death. Tumour antigen-specific T cell activation and recruitment result from this which is enhanced by immunologic recall to intratumoral replicating virus from previous vaccination. Type I/III interferon-dominant inflammation follows (Sloan et al., 2021).

### 5.7 Combination therapy

Numerous pharmacological compounds that are known to affect the Tumor micro environment or have an impact on GB cells are currently being used in clinical practice for a variety of illnesses. Small-molecule inhibitors are intended to prevent tumour growth, survival, angiogenesis, and metastasis by blocking signalling pathways. In clinical trials, tyrosine kinase inhibitors (TKIs) have been evaluated as monotherapies for individuals with GB; nevertheless, their therapeutic impact has been limited (Kim and Ko, 2020). TKI and CAR T cell combinations have demonstrated synergistic results in the treatment of other solid cancer types, making them a good option to consider in GBM patients (Wu et al., 2019). A chemical involved in cell-cell adhesion called protein phosphatase 2A (PP2A) is inhibited by the tiny molecule LB-100. Combination with LB-100 can improve the effectiveness of CAIX-specific CAR T cell therapy in both *in vivo* and *in vitro* GBM models (Brown et al., 2016; Cui et al., 2019).

Combination of DNX-2401 and TMZ for newly diagnosed GB, temozolomide (TMZ), an alkylating drug, is a part of the conventional therapy regimen. Promoter methylation is known to play an epigenetic role in TMZ chemosensitivity by suppressing O6-methylguanine-DNA methyltransferase (MGMT) (Dunn et al., 2009).Pre-clinically, it was demonstrated that DNX-2401 infection decreased the IC50 of TMZ and MGMT levels in human GBM cell lines (U87 and T98G) *in vitro* (Alonso et al., 2007). Combination treatment with DNX-2401 and TMZ greatly increased the length of survival in an immunodeficient mice model of GBM xenograft. Later research by Kleijn et al. demonstrated that the combination was similarly effective in immunocompetent mouse models of GBM.These investigations served as justification for the DNX-2401 and TMZ combination therapy Phase I trial, which is currently taking place in Spain for recurrent glioblastoma (Kiyokawa and Wakimoto, 2019).

For a long time, researchers have been looking at using adenovirus vectors carrying the HSV thymidine kinase gene (HSV-tk) as a treatment for GBM. When a nucleoside analogue prodrug, like ganciclovir, is administered systemically to tumour cells after adenoviral transduction of HSV-tk, it phosphorylates the prodrug, stops DNA replication, and causes cell death in proliferating tumour cells as well as nearby cells through the bystander effect. Tumour neoantigens are released when cancer cells die, and this triggers cellular immune responses that fight the malignancy (Barba et al., 1994; Perez-Cruet et al., 1994). Table 2.

**TABLE 2:** List of Therapeutic agents and their targets in treating GB.

Therapeutics	Mode of action	Treatment effect	Studied in	Clinical trials	Author
Adaptive T cells					
IL13Rα2-CAR-T cells	Targets CAR T cells possessing a mutated form of IL-13 in the CAR construct (Thaci et al., 2014)	Feasible and safe with clinical safe reported in human pilot study (Brown et al., 2015)	Humans	phase I safety and feasibility trial (Brown et al., 2022)	Thaci et al. (2014)
					Brown et al. (2015)
					Brown et al. (2022)
cytokine-induced killer (CIK) Immunotherapy	Cytokine-induced killer cells therapy for newly diagnosed GB. (Kong et al., 2017)	Neutropenia events, pneumonia and aute renal failure (Kong et al., 2017)	Humans	Phase III RCT (Kong et al., 2017)	Kong et al. (2017)
Checkpoint Inhibitor					
Pembrolizumab	Anti-programmed death 1 (PD-1) immune checkpoint inhibitor (Reardon et al., 2021)	Rash, proteinuria, fatigue, increased alanine aminotransferase, and hypertension (Reardon et al., 2021; Sahebjam et al., 2021)	Humans	phase II RCT (Nayak et al., 2021)	Reardon et al. (2021); Sahebjam et al. (2021), Lakshmi N, et al., 2021
Nivolumab	Antibody targeting the programmed death-1 (PD-1) immune checkpoint receptor. (Reardon et al., 2020)	Fatigue, Increased alanine aminotransferase, Headache, Increased lipase, Pulmonary embolism (Reardon et al., 2020)	Humans	phase III RCT (Reardon et al., 2020)	Reardon et al. (2020)
Ipilimumab	Checkpoint inhibitor acting against the CTLA-4 protein (Youssef and Dietrich, 2020)	fatigue and diarrhea (Youssef and Dietrich, 2020)	Humans	FDA approved (Brown et al., 2020)	Youssef Dietrich (2020)
					Brown et al. (2020)
Bevacizumab	Binds to vascular endothelial growth factor A (VEGF-A) and prevents its interaction with VEGF receptor tyrosine kinases VEGFR1 and VEGFR2 on the surface of endothelial cells, (Ferrara et al., 2004)	reduced interstitial edema and interstitial hypertension (Ferrara et al., 2004)	Humans	FDA approved (Wu et al., 2021)	Ferrara et al. (2004), Wu et al. (2021)
Vaccine					
Rindopepimut	Targets treatment of EGFRvIII-positive GB. (Weller et al., 2017)	fatigue, rash, nausea, pruritus, and headache (Weller et al., 2017)	Humans	phase III RCT (Schuster et al., 2015)	Weller et al. (2017)
					Schuster et al. (2015)
Signaling Pathway targets					
9 - oxo mic. heliolide	Inhibitor of NF-κB and STAT3 signaling pathways, along with induction effects of cell apoptosis. (Sahebjam et al., 2021)	Not known	Mice	Not known	Zeng et al. (2021)
Erlotinib	reversible competitive inhibitor of the adenosine triphosphate region of the EGFR tyrosine kinase domain (De Groot et al., 2008)	rash, fatigue and diarrhea (De Groot et al., 2008)	Humans	phase II study (De Groot et al., 2008)	de Groot et al. (2008)
NSAIDs— diclofenac and celecoxib	Wnt signaling inhibitor (Sareddy et al., 2013)	Not known	Humans	More prospective investigation of the effect of NSAID is required (Bruhns et al., 2018)	Sareddy et al. (2013)
					Bruhns et al. (2018)
Dexamethasome	Cell cycle progression Inhibitor through down-regulating of cyclin D1 and inhibition of ERK1/2 phosphorylation. (Liu et al., 2009; Kostaras et al., 2014)	myopathy, abnormal glucose metabolism, gastrointestinal complications, irritability, anxiety, insomnia, pneumonia infection (Swildens et al., 2022)	Humans	Not known	Liu et al. (2009)
	Reduces number of macrophages and T cells in GB microenvironment. Dexamethasone removes checkpoint inhibitor-induced antitumor effects in glioblastoma. (Burns et al., 2016)				Swildens et al. (2022)
					Kostaras et al. (2014)
Cilengitide (CGT)	Integrin inhibitor that targets αvβ3 and αvβ	No significant adverse events, and no patients developed intracranial hemorrhage (Zhao et al., 2016)	Humans	phase II study (195)	Zhao et al. (2016)
					Reardon et al. (2021)

## 6 Conclusion

Despite considerable progress in the understanding of molecular mechanisms and signaling molecules that regulate stemness, invasion and proliferation in the GB micro-environment, the prognosis still remains poor resulting in high recurrence and mortality. This requires further research on identification of upstream targets that trigger conversion of normal cells to cancer phenotypes. Since the downstream regulators of GB like VEGF or immune responses have a constitutive role at the system level, scope of intervention is limited. Identification of molecular targets and mechanisms that are exclusive to GB and its micro-environment holds the key to effective treatment modalities. With the advent of personalized medicine, the therapeutic strategies also need to be upgraded to individual centric rather than population centric approaches. Considering the wide variability in pro-carcinogenic factors amongst patients, personalized approaches could probably result in better patient outcome (Buckley, 1929; Sachs, 1954; Jelsma and Bucy, 1967; Edland et al., 1971; Ramsey and Brand, 1973; Rosenblum et al., 1973; Tani et al., 1973; Marton et al., 1974; Yung et al., 1976; Heiss, 1978; Morantz et al., 1979; Neuwelt et al., 1980; Otsuka et al., 1984; Jacobs et al., 1986; Ellemann et al., 1988; Plate et al., 1992a; Shweiki et al., 1992; MURATA et al., 1993; Tada et al., 1994; Miescher et al., 1997; Liau et al., 2000; Hao et al., 2002; Rodrigues et al., 2010; Reardon and Cheresh, 2011).
